# Upregulation of CFTR in patients with endometriosis and its involvement in NFκB-uPAR dependent cell migration

**DOI:** 10.18632/oncotarget.16441

**Published:** 2017-03-22

**Authors:** Wenqing Huang, Aihong Jin, Jieting Zhang, Chaoqun Wang, Lai Ling Tsang, Zhiming Cai, Xiaping Zhou, Hao Chen, Hsiao Chang Chan

**Affiliations:** ^1^ Epithelial Cell Biology Research Center, School of Biomedical Sciences, Faculty of Medicine, The Chinese University of Hong Kong, Shatin, PR China; ^2^ Institute of Biomedical and Pharmaceutical Sciences, Guangdong University of Technology, Guangzhou, PR China; ^3^ Sichuan University – The Chinese University of Hong Kong Joint Laboratory for Reproductive Medicine, West China Second Hospital, Sichuan University, Chengdu, PR China; ^4^ Department of Gynecology, The Second People’s Hospital of Shenzhen, Shenzhen, PR China

**Keywords:** endometriosis, CFTR, NFκB signaling, uPAR, cell migration

## Abstract

Endometriotic tissues exhibit high migration ability with the underlying mechanisms remain elusive. Our previous studies have demonstrated that cystic fibrosis transmembrane conductance regulator (CFTR) acts as a tumor suppressor regulating cell migration. In the present study, we explored whether CFTR plays a role in the development of human endometriosis. We found that both mRNA and protein expression levels of CFTR and urokinase-type plasminogen activator receptor (uPAR) were significantly increased in ectopic endometrial tissues from patients with endometriosis compared to normal endometrial tissues from women without endometriosis and positively correlated. In human endometrial Ishikawa (ISK) cells, overexpression of CFTR stimulated cell migration with upregulated NFκB p65 and uPAR. Knockdown of CFTR inhibited cell migration. Furthermore, inhibition of NFκB with its inhibitors (curcumin or Bay) significantly reduced the expression of uPAR and cell migration in the CFTR-overexpressing ISK cells. Collectively, the present results suggest that the CFTR-NFκB-uPAR signaling may contribute to the progression of human endometriosis, and indicate potential targets for diagnosis and treatment.

## INTRODUCTION

Endometriosis is a common benign gynecological disorder defined as the presence of endometrial tissue outside the endometrium and myometrium, and affects almost 10% of women at reproductive age, even 35-50% of women with pelvic pain and/or infertility [1, 2]. Surgery is the most common treatment, but it recurs at a rate of 40% in 5 years [3]. The ectopic endometrium acquires the ability to invade and attach the peritoneum by degrading extracellular matrix and recruiting nerves/blood vessels into the lesions [4, 5]. Although endometriosis is a benign disease, the migratory/invasive potential of ectopic endometrial cells is comparable to that of cells from a metastatic cancer [6].

Urokinase type plasminogen activator (uPA) and its receptor (uPAR) are well-documented to be involved in multiple tissue remodeling and cancer metastasis [7, 8]. Plasminogen was activated and converted to plasmin by the binding of uPA and uPAR, initiating pericellular proteolysis and cell migration and contributing to cancer metastasis [9]. Apart from its role in cancers, uPA signaling was also involved in uterine physiology and the initiation of menstruation [10, 11]. It has been reported that progesterone inhibits the activation of uPA *via* increase of PAI-1 and uPAR expression in endometrial stromal cells [12]. Of note, the expression of uPA and soluble uPAR has been observed to be upregulated in the endometrium of women with endometriosis [13, 14], suggesting the involvement of the uPA system in the progression of endometriosis. However, the molecular mechanism regulating uterine uPAR remains unclear although its regulation by NFκB is well-known in many other tissues [15, 16].

CFTR is a cAMP-activated Cl^−^- and HCO_3_^−^-transporting channel, expressed in epithelial cells of various organs [17]. Mutations of CFTR result in cystic fibrosis (CF), a lethal genetic disease with multi-organ defects, including infertility [18, 19]. In the female reproductive tract, CFTR is expressed in the vagina, cervix, uterus, fallopian tube and ovary [20–22]. During the estrus cycle, the expression of CFTR in mouse endometrium is upregulated by estrogen [23] and downregulated by progesterone [24]. This cyclic change of CFTR expression pattern is thought to be important for regulating electrolyte transport and uterine fluid environment required for sperm transport, capacitation and embryo implantation [21, 25]. Apart from its channel function, CFTR has also been shown to be involved in multiple cellular processes including cancer development and metastasis [26–28]. Our previous studies have demonstrated that CFTR acts as a tumor/EMT suppressor, which is shown to be downregulated and associated with poor prognosis in prostate cancer [16], breast cancer [26], colon cancer [29] and lung cancer [30]. However, opposite role of CFTR has been observed in the female reproductive tract. A recent study on ovarian cancer found that CFTR level was significantly increased compared to the benign ovarian tumor and normal ovaries and its expression level was significantly associated with advanced FIGO stage [31]. It has also been demonstrated that the expression of CFTR is significantly increased and highly associated with cervical cancer progression, aggressive behaviors and poorer prognosis [32], accompanied by elevated expression of NFκB p65 [33]. Furthermore, our recent study has demonstrated a direct interaction between CFTR and NFκB p65 in human epithelial colorectal adenocarcinoma cells by Co-Immunoprecipitation [34]. Of interest, CFTR has been implicated in the pathogenesis of endometriosis by a proteomic study together with 20 proteins identified [35]. However, quantified expression level of CFTR in human endometriosis has not been confirmed and its exact role in the development of endometriosis remains unexplored. The upregulated expression of CFTR in female tract cancers and its interaction with NFκB, and the fact that uPAR is positively regulated by NFκB singling [15], led us to the hypothesis that CFTR might be involved in the progression of endometriosis by promoting NFκB and uPAR-dependent cell migration. To test this hypothesis, we thus undertook the present study to examine the expression and correlation of CFTR and uPAR in ovarian ectopic endometrioic samples and compared to normal endometria in human. The role of CFTR and its signaling involved in cell migration in a human endometrial cell line was also investigated.

## RESULTS

### Significantly upregulated CFTR and uPAR expression in ovarian ectopic endometria

**Figure 1:**
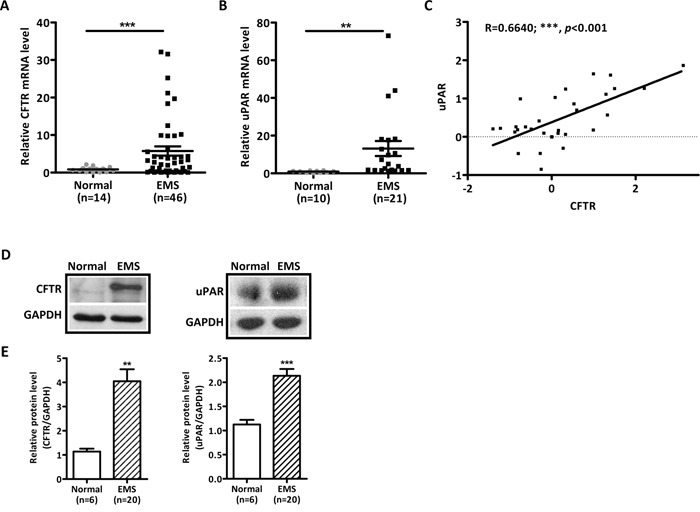
Abnormally high expression levels and strong correlation of CFTR and uPAR in human ovarian ectopic endometriotic tissues **(A)** mRNA level of CFTR in human ectopic endometrium (EMS, n = 46) and normal endometrium from women without EMS (Normal, n = 14) was determined by qPCR. ***, *p* < 0.001, t-test. **(B)** qPCR results of uPAR expression in remaining available human ectopic endometria (EMS, n = 21) and normal endometria from women without EMS (Normal, n = 10). **, *p* < 0.01. **(C)** Correlation analysis between CFTR and uPAR in both CFTR and uPAR checked samples. The expression levels of uPAR are positively correlated with the CFTR expression levels in human endometriotic tissues with the r = 0.6640 (***, *p* < 0.001). **(D)** Representive western blot showing protein expressions of CFTR and uPAR in EMS and normal endometria. GAPDH was used as the loading control. **(E)** Statistical analysis of CFTR and uPAR protein levels in EMS (n = 20) compared to normal endometrium (n = 6). **, *p* < 0.01, ***, *p* < 0.001. Values represent mean ± SEM.

We first evaluated the mRNA expression of CFTR in 46 of ovarian endometriotic lesions and 14 of normal endometria from the infertile patients without endometriosis. Quantitative real-time PCR (qPCR) results demonstrated that the expression of CFTR in endometriotic lesions was significantly higher than that in normal endometria (Figure 1A). In the remaining available samples, 21 endometriotic lesions and 10 normal endometria, we also examined the expression of uPAR and found significantly higher expression levels in endometriotic lesions compared to that of normal endometria (Figure 1B). Correlation analysis of both CFTR and uPAR in the same pool of samples revealed that the mRNA levels of CFTR were positively correlated with uPAR expression levels (Figure 1C, r = 0.6640, ***, *p* < 0.001). Consistent with mRNA expression, the protein levels of CFTR and uPAR were significantly increased in the ovarian endometriotic lesions (Figure 1D and 1E).

### CFTR expression regulates the migration of ISK cells

**Figure 2:**
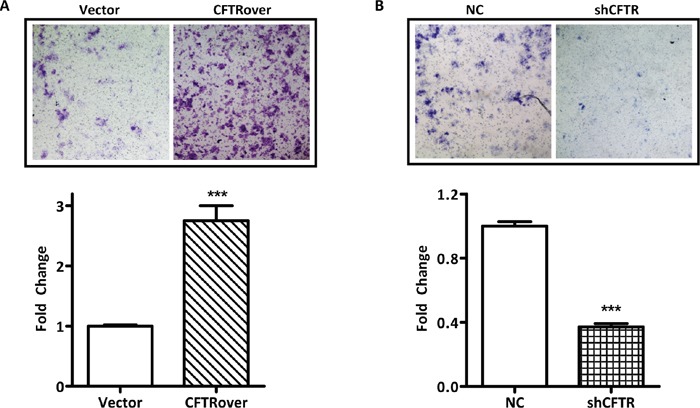
CFTR promotes migration of ISK cells **(A)** Cells transfected with full-length CFTR plasmid (CFTRover) significantly increased cell migration compared with vector control group (Vector) in the transwell migration assay, ***, *p* < 0.001. **(B)** Cells infected with CFTR shRNA (shCFTR) suppressed cell migration compared with control group (NC), ***, *p* < 0.001. The experiments were repeated for 3 times. Values represent mean ± SEM.

Next, we investigated whether endometrial cell migration may be affected by the levels of CFTR expression. We first performed migration assay in ISK cells treated with a CFTR inhibitor (inh172). Surprisingly, the migration rate was not changed in the inh172 treated group in comparison to that in blank and DMSO control (Supplementary Figure 1). Given that ISK is cancer origin, we further examined the effect of CFTR inhibition on migration in primary culture of mouse endometrial epithelial cells and observed similar results showing insignificant effect with CFTR inhibition (Supplementary Figure 1), suggesting that the ion channel function of CFTR might not be involved in the regulation of cell migration in endometrial cells. Therefore, we further examined the migration ability in ISK cells with CFTR overexpression or knockdown using transwell migration assay. As shown in Figure 2A, overexpression of CFTR resulted in enhanced cell migration after 48-hour incubation compared to that of the vector control. As expected, knockdown of CFTR led to reduced cell migration compared to that of the negative control (Figure 2B). These results suggest that CFTR expression levels, but not its channel activity, are tightly coupled to the migratory potential of endometrial cells.

### Overexpression of CFTR increases cell migration through activation of NFκB and uPAR signaling

**Figure 3:**
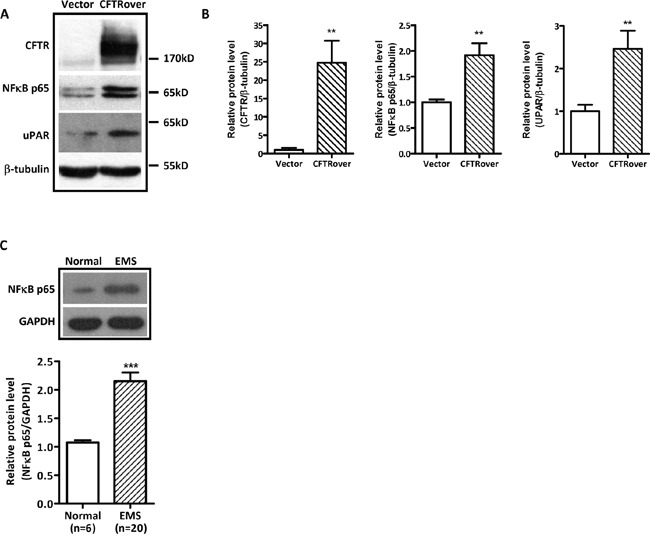
Effect of CFTR overexpression on NFκB p65 and uPAR expression in ISK cells **(A)** Western blot analysis of upregulation of NFκB p65 and uPAR in CFTR-overexpressing ISK cells. **(B)** The corresponding statistical analysis (**, *p* < 0.01 *vs* vector control), the experiments were repeated 6 times. Values represent the mean ± SEM. **(C)** Western blot analysis of NFκB p65 in human ectopic endometrium (n = 20) and normal endometrium (n = 6). ***, *p* < 0.001, t-test. Values represent mean ± SEM.

**Figure 4:**
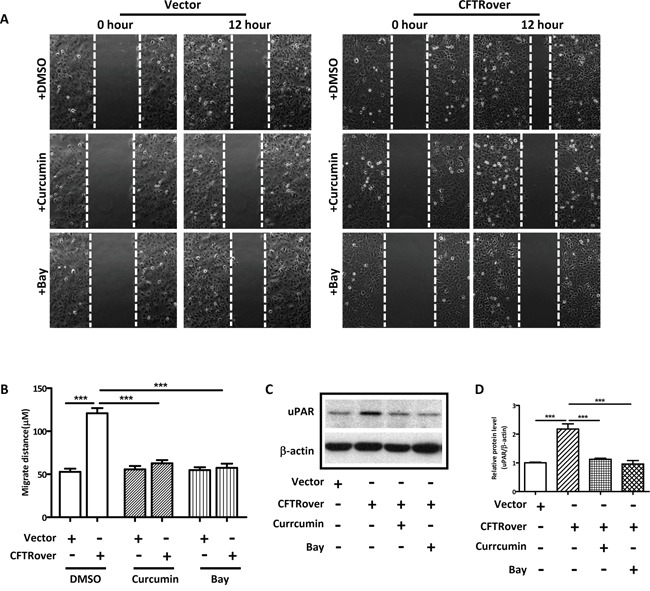
NFκB inhibitors reduce cell migration and uPAR expression in CFTR-overexpressing ISK cells **(A)** Representative images of treated and untreated CFTR-overexpressing ISK cells are presented (×10 magnification). ISK cells transfected with vector control or full-length CFTR were treated with NFκB inhibitors curcumin (10 μM) or Bay (2 μM) for 24 hours. **(B)** Statistical analysis of migration ability of ISK cells. Values represent the mean ± SEM (***, *p* < 0.001 *vs* DMSO). **(C)** Western blot analysis of uPAR treated with NFκB inhibitors curcumin (10 μM) or Bay (2 μM) in CFTR-overexpressed ISK cells. **(D)** The corresponding statistical analysis (***, *p* < 0.001), the experiments were repeated 3 times. Values represent the mean ± SEM.

Given the presently observed correlation of CFTR and uPAR expression in human endometriosis and the well-established role of uPAR in cell invasion/migration [36, 37] and its regulation by NFκB [15], we explored the involvement of NFκB in mediating the effect of CFTR on uPAR expression and cell migration. We overexpressed CFTR in ISK cells, and as expected, the expression levels of both NFκB p65 and uPAR were significantly increased in the CFTR-overexpressing ISK cells (Figure 3A and 3B). We also examined NFκB expression levels in some of the human endometriotic tissues (n = 20), which exhibited high levels of CFTR and uPAR (Figure 1), and detected significantly higher levels of NFκB expression compared to the normal control (n = 6), consistent with the finding from ISK cells (Figure 3C). Furthermore, the migration of the CFTR-overexpressing ISK cells was significantly inhibited when treated with NFκB inhibitor Curcumin (10μM) or Bay (2μM) (Figure 4A and 4B). Western blot results showed that both curcumin and Bay treatment significantly reduced the expression of uPAR in CFTR overexpressing ISK cells (Figure 4C and 4D), indicating the involvement of NFκB in mediating the CFTR effect on uPAR expression, and thus cell migration.

## DISCUSSION

This is the first quantitated expression study of CFTR in human endometriotic tissues and functional study in human endometrial cells establishing the important role of CFTR in cell migration. A strongly positive correlation between CFTR and uPAR is found in ovarian ectopic endometriosis, suggesting a link between CFTR and uPAR. The CFTR effect on uPAR expression is demonstrated to be mediated by NFκB. While uPAR has been shown to be regulated by NFκB in various cancers [15, 38], this study is the first to demonstrate the involvement of NFκB in the regulation of uPAR expression by CFTR in human endometrial cells since NFκB inhibitors can reverse the CFTR overexpression-induced upregulation of uPAR. Thus, the upregulated CFTR appears to result in abnormally high expression of uPAR through NFκB, and this explains the strongly positive correlation between CFTR and uPAR observed in the human endometriotic tissues. It should be noted that our previous studies have also observed a link between CFTR and uPAR in prostate cancer and non-small cell lung cancer [16, 30], however, CFTR was found to suppress uPAR in the cancer tissues in contrast to a positive correlation with uPAR observed in the present study. The CFTR-dependent signaling pathways involved in the cancers studied and endometriosis appear to be different.

The present study has demonstrated the involvement of NFκB in mediating the effect of CFTR in endometrial cells. A link between CFTR and NFκB is well documented although the exact relationship remains obscure and controversial. An inverse relationship between the two is well established in CF, where chronic inflammation in the lung, a hallmark of the disease, is attributed to the upregulation of NFκB due to CFTR mutations [39, 40]. The inverse relationship between CFTR and NFκB has also been found in male reproductive tract, where high temperature-downregulated CFTR with upregulated NFκB/COX2/PGE2 was found to be responsible for impaired testis-blood barrier, and thus disrupted spermatogenesis as seen in cryptorchidism [41]. However, a positive link between CFTR and NFκB has been found in the embryo [27] and cervical cancer [33]. Our present results have also demonstrated a positive relationship between CFTR and NFκB in ISK cells (Figure 3). Similar to the finding from previous reports [27, 33], the present study also detected upregulated NFκB in the human endometriotic lesions exhibiting high levels of CFTR and uPAR, confirming a positive relationship between CFTR and NFκB in endometriosis. Taken together, it appears that CFTR may positively or negatively regulate NFκB, depending on cellular contexts. The detail mechanism underlying the cell type-specific regulation of NFκB by CFTR awaits further investigation.

The present study has also demonstrated a functional role of CFTR in endometrial cell migration. Interestingly, the CFTR-regulated cell migration is not dependent on its ion channel function but its expression level. This suggests that CFTR does not merely function as an ion channel. As a membrane protein, CFTR has been shown to interact with quite a number of proteins [29, 42, 43], which may be independent of its channel function. The present results indicate that aberrantly high expression of CFTR, but not its channel function, and abnormally high uPAR expression confer a propensity of exaggerating cell migration that leads to the progression of endometriosis. Although the exact mechanism underlying the regulatory effect of CFTR on uPAR awaits further investigation, the present finding offers a potential diagnostic strategy for endometriosis targeting both CFTR and uPAR.

Human endometriosis is considered an estrogen-dependent disorder [44, 45] and the currently offered clinical treatments include surgical and hormonal treatments to alleviate pain and infertility [46, 47]. The presently demonstrated high expression of CFTR in ovarian ectopic endometriotic tissues can be explained by the well-established expression regulation of CFTR by estrogen [23, 48]. The demonstrated role of CFTR in cell migration, together with dependence of CFTR expression on estrogen, provides a novel insight into the pathogenesis of endometriosis. In other words, the pathological progression of endometriosis may be triggered by abnormal estrogen-stimulated CFTR expression in the endometrium. The present findings also provide a rationale for the current treatment strategies for endometriosis. Hormonal treatments, such as hormonal contraceptives, are in place mostly for their anti-estrogen effects [45, 47], which are known to downregulate CFTR. Previous studies have shown that CFTR is cyclically expressed in mouse uterus [49], upregulated by estrogen [23] and downregulated progesterone [24]. Therefore, the clinically offered hormonal treatments for endometriosis could be mediated by the hormonal effect on CFTR downregulation. This is consistent with the important role of CFTR in cell migration and thus progression of endometriosis demonstrated in the present study. It should be noted that hormonal treatments have limitations for their side effects [47, 50]. The present finding, therefore, suggests that targeting CFTR or its signaling (such as NFκB and uPAR) may be attractive alternatives for the intervention of endometriosis. The present findings warrant future investigation of diagnostic and treatment strategies for endometriosis targeting CFTR and related signaling.

## MATERIALS AND METHODS

### Human tissue collection

Forty-six women with endometriosis and fourteen women without endometriosis but infertility were recruited to the present study according to the previous criteria [51]. Endometriotic tissues were collected from the walls of endometriomas in patients with ovarian ectopic endometrium, and normal endometrial tissues (confirmed by pathologic diagnosis) were collected from normal endometrium by curettage in patients with infertility. All tissues were taken at the proliferative phase of the menstrual cycle.

### Ethical approval

All samples were collected with informed consent from each patient and approval from the ethics committee of the Second People’s hospital of Shenzhen (201306015) in China.

### Cell culture, gene overexpression and knockdown

Primary mouse uterine epithelial cells were isolated and cultured as previously described [52]. Human endometrial Ishikawa cells (ISK), a highly differentiated endometrial epithelial adenocarcinoma cell and widely used in the studies of endometriosis [53–55], was cultured in Roswell Park Memorial Institute (RPMI)-1640 supplemented with 10% FBS and 1% Penicillin-Streptomycin in 5% CO_2_ incubators at 37°C. The pEGFP plasmid expressing wild-type CFTR (pEGFP-CFTR) was kindly provided by Professor Tzyh-Chang Hwang (University of Missouri-Columbia). For overexpression of CFTR, ISK cells were seeded in 6-well plates at 4 × 10^5^ cells/well. 2.5 μg of pEGFP-CFTR or pEGFP were transfected using 6 μl Lipofectamine 2000 (Invitrogen, USA) following the manufacturer's instructions. For CFTR knockdown, lenti-virus (LV3) packaged shRNAs-targeting human CFTR (5’-GAA GTA GTG ATG GAG AAT GTA-3’) or scrambled noncoding shRNAs (5’-TTC TCC GAA CGT GTC ACG TTT-3’) were purchased from GenePharma (Shanghai, China). The viruses (1×10^9^ TU/ml) were transduced into ISK cells with polybrene (5μg/ml). Cells were cultured in the presence of puromycin (5 μg/ml) for three passages to select stable clones before migration assay.

### RNA isolation, reverse transcription, and quantitative real-time PCR

Total RNA was extracted from human samples and cells by Trizol reagent (Invitrogen, USA) according to the instructions. Complementary DNA (cDNA) was synthesized from 1 μg RNA with iScript cDNA synthesis kit (Bio-Rad, USA). For quantitative PCR, assays were performed in triplicate on an Applied Biosystems 7500Fast Real-Time PCR System with Taqman primers of CFTR (Hs00357004_m1) and uPAR (Hs00182181_m1) purchased from Applied Biosystems (USA).

### Western blot

Human samples and ISK cells were lysated in a lysis buffer (RAPI buffer: 50 mM Tris-HCl, pH8.0, 150 mM NaCl, 1% NP-40, 0.5% Sodium deoxycholate, 0.1% SDS). Western blot analysis was performed as described in our previous study [56]. Equal amounts of protein were subjected to 8% SDS-PAGE and were transferred onto nitrocellulose membranes. The transferred membrane was blocked with 5% skim milk in TBST (50 mM Tris-HCl, 150 mM NaCl, and 0.05% Tween 20, pH 8.0) for 1 h. The membrane was then incubated with the primary antibody in TBST plus 2% fat-free milk at 4°C for overnight. Antibodies used in this study were: Anti-CFTR (Almone Lab, catalog No.: ACL-006, 1:500), anti-uPAR (Santa Cruz, catalog No.: sc-10815, 1:500), anti-NFκB p65 (Cell signaling, catalog No.: 9496, 1:500), anti-GAPDH (Santa Cruz, catalog No.: sc-47724, 1:5000), anti-β-tubulin (Santa Cruz, catalog No.: sc-9104, 1:2000), anti-β-actin (Sigma, catalog No.: A2066, 1:5000). The membrane was subsequently washed with TBST and incubated for 1 hour with peroxidase-conjugated secondary antibody. The membrane was washed 3 times with TBST and then detected by chemiluminescence (Amersham, Piscataway, NJ).

### Migration assay

Migration assay was used to assess the effect of CFTR on migration. Two different migration assays were performed in the present study. Briefly, the transwell migration assay was carried out in transwell champers with 8 μm Pore Polycarbonate Membrane Inserts (Corning Incorporated, MA, USA). A total of 4 × 10^4^ cells were seeded into the upper compartment of the transwell chambers in RPMI-1640 medium without FBS. Medium plus 20% FBS (600 μl/chamber) was added to the lower compartment. After 48-hour incubation, the cells in the upper side of the chambers were removed using tissue paper, then the migrated cells attached to the lower surface of the filter were fixed with 4% PFA, stained with 0.5% crystal violet solution (w/v), and photographed under a phase contrast microscope and then counted cells in 5 randomly selected microscopic fields for statistical analysis.

For the wound healing migration assay, 5 × 10^5^ cells were cultured in a 6-well plate and scratched with a 10 μl culture tip in the fresh medium (RPMI-1640+1% FBS) with or without NFκB signaling inhibitor curcumin (Sigma, catalog No.: C1386, USA) or Bay (Cayman, catalog No.:10010266, USA). The closure of the wound via the migration of cells into the wound was tracked and recorded using a live imaging system (Carl Zeiss, Jena, Germany) at 1 hour intervals for 24 hours. Cell migration was determined by measuring distances between parallel lines of cells. At least five imaging views were calculated on each well to quantify the migration rates.

### Statistical analysis

All morphometric data were collected blindly. The results are shown as mean ± S.E.M. Statistical significance for comparison between two measurements was determined by t test. One or two-way ANOVA was used for evaluation of the 3 or more measurements. Correlation analysis was carried out using Pearson's correlation for normally distributed data. All statistical analyses were performed using Prism 6.0 (GraphPad Prism). Differences were considered to be statistically significant at *p* < 0.05.

## SUPPLEMENTARY FIGURE


