# Supplementary material for: Upregulation of CFTR in patients with endometriosis and its involvement in NFκB-uPAR dependent cell migration
Source: Oncotarget. 2017 Mar 22;8(40):66951–9. doi: 10.18632/oncotarget.16441 (PMC5620148; doi:10.18632/oncotarget.16441)
Supplement: Supplementary file 1 [file oncotarget-08-66951-s001.pdf]

Upregulation of CFTR in patients with endometriosis and its involvement in NFκB-uPAR dependent cell migration

SUPPLEMENTARY FIGURE

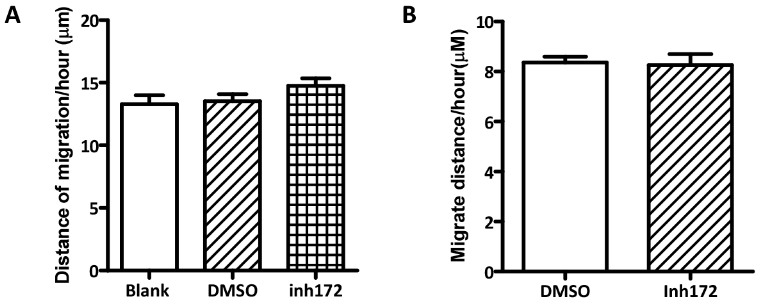

Supplementary Figure 1: Insensitivity of cell migration to CFTR inhibitor in primary cultured mouse endometrial epithelial cells and ISK cells. Primary cultured mouse endometrial epithelial cells **(A)** and ISK cells **(B)** were treated with 10 μM inh172 for 24 hours. Statistical analysis of migration ability of mouse endometrial epithelial cells and ISK cells. Values represent the mean ± SEM.
